# Remaining Useful Life (RUL) Prediction of Equipment in Production Lines Using Artificial Neural Networks

**DOI:** 10.3390/s21030932

**Published:** 2021-01-30

**Authors:** Ziqiu Kang, Cagatay Catal, Bedir Tekinerdogan

**Affiliations:** 1 Information Technology Group, Wageningen University & Research, 6706 KN Wageningen, The Netherlands; ziqiu.kang@wur.nl; 2 Department of Computer Science and Engineering, Qatar University, Doha 2713, Qatar; ccatal@qu.edu.qa

**Keywords:** machine learning, production lines, predictive maintenance, data mining, maintenance prediction

## Abstract

Predictive maintenance of production lines is important to early detect possible defects and thus identify and apply the required maintenance activities to avoid possible breakdowns. An important concern in predictive maintenance is the prediction of remaining useful life (RUL), which is an estimate of the number of remaining years that a component in a production line is estimated to be able to function in accordance with its intended purpose before warranting replacement. In this study, we propose a novel machine learning-based approach for automating the prediction of the failure of equipment in continuous production lines. The proposed model applies normalization and principle component analysis during the pre-processing stage, utilizes interpolation, uses grid search for parameter optimization, and is built with multilayer perceptron neural network (MLP) machine learning algorithm. We have evaluated the approach using a case study research to predict the RUL of engines on NASA turbo engine datasets. Experimental results demonstrate that the performance of our proposed model is effective in predicting the RUL of turbo engines and likewise substantially enhances predictive maintenance results.

## 1. Introduction

A production line is typically a set of equipment or machines established in a factory where components are assembled sequentially to make a finished product [1]. Nowadays, manufacturers use different kinds of machines for production, and over time, these machines and the associated equipment may deteriorate, and sometimes even the entire production line may fail [2]. Breakdowns seriously impact the performance and cost of production lines and often lead to a dramatic reduction of availability because of the costly maintenance period [3].

For avoiding these failure cases, the maintenance of resources is often planned in advance [4]. However, the maintenance cost of some industries can increase by up to 70% of the total cost [5]. As such, the reduction of maintenance costs is considered a crucial and substantial advantage to the manufacturer in a highly competitive manufacturing sector, such as, for example, the semiconductor industry. In many industrial sectors such as automotive manufacturing, maintenance management is an explicit strategic issue to take necessary actions on time [6,7]. Fixing the production line after the breakdown can be more costly than conducting preventive maintenance ahead of the breakdown [8,9]. Also, the revision of the production plan can cause variability in service and product quality [1].

One of the approaches to reducing maintenance costs is known as preventive maintenance. Preventive maintenance can be considered as a kind of proactive approach, which systematically inspects and maintain the equipment to avoid breakdowns. There exist several factors that can complicate the maintenance operation of production lines, such as system configurations, cost of maintenance resources, degradation profiles of machines, maintenance schedule, and recent status of machines [10].

From a machine learning perspective, there are several challenges of preventive maintenance in production lines. First, it is indeed difficult to acquire machine malfunction data and label the failure case in practice in the datasets. Second, there exists a huge amount of process data (i.e., big data) generated in production lines, and processing of this big data requires a special infrastructure, expert knowledge, and custom smart software. Last, many companies do not share this type of data publicly due to data privacy, and as such, researchers in this field are unable to validate new models with more datasets. To this end, there is a need for further research to come up with effective measures and new models in order to implement predictive maintenance effectively in production lines. 

Remaining useful life (RUL) is a key metric and critical to predicting the failure of a machine in the production line. The challenge of RUL prediction is that RUL is not mostly labeled in the training dataset, and therefore, supervised learning algorithms of machine learning cannot be applied in this case. A health index needs to be correctly defined and interpolated to map the relationship between features and the RUL. After this interpolation step, a machine learning-based model can be used to predict the health index by learning the interpolated data accurately. The machine learning-based approach is expected to handle the adverse impacts of noise in the dataset and possible sensor problems (i.e., sensor drift) that might arise during the operation of the production line.

The main objective of our study is to minimize the adverse effects of breakdowns and build a novel machine learning-based RUL prediction model. We propose and validate a new machine learning-based model in predicting the failure of equipment (i.e., RUL prediction) in production lines and analyze the applicability of machine learning algorithms in predicting the failure of equipment in product lines. Specifically, we focus on jet engines and use the run-to-failure data of similar jet engines to predict the failures of jet engines. This data includes several measurements including temperatures, pressures, rotating speeds of jet engines. During our experiments, different machine learning algorithms, pre-processing and feature selection techniques, and parameter optimization approaches are investigated to build a novel model to predict the risk of production line failure.

The concept of RUL is used to evaluate the risk of production line breakdown. For the case study, the NASA dataset on turbo engines has been used in this study [11]. The proposed model developed in this case study can also be applied to the other production lines. The case study demonstrates the effectiveness of our prediction model to predict the RUL within the scope of predictive maintenance.

The contributions of this study are two-fold, which are listed as follows:

1. We developed a novel RUL prediction approach that utilizes the principal component analysis (PCA) feature selection algorithm, grid search parameter optimization algorithm, and multi-layer perceptron (MLP) machine learning algorithm.
2. Since the RUL was not provided in training datasets, a polynomial function was fitted to HIs, and the interception between the polynomial and cycle axis was calculated as the failure point.

The following sections of this article are organized as follows: Section 2 explains the background and related work. Section 3 presents the data analysis. Section 4 describes the methodology, and Section 5 explains the experimental results. Section 6 presents the discussion and threats to validity. Section 7 presents conclusions and future work.

## 2. Background and Related Work

Four categories of maintenance policies have been suggested in the literature [12]: run-to-failure (R2F), preventive maintenance (PM), condition-based maintenance (CBM), and predictive maintenance (PdM). R2F is executed after the failure, and as such, this is the simplest approach and the costliest one, among others. PM is performed periodically based on a schedule. CBM checks several conditions, and if one of them indicates that there is a degradation of the equipment, CBM is directly executed. PdM (a.k.a., statistical-based maintenance) applies predictive analytics and tools to determine the required actions. As in the case of CBM, PdM also performs the maintenance only when it is necessary. Several authors [12,13] emphasize that CBM and PdM are actually addressing the same maintenance policy, and as such, they do not distinguish between them.

Many modeling approaches have been used in literature for maintenance policy analysis and reliability engineering [2]. Some of these approaches are Markov chains [14,15], Petri nets [16], fault tree analysis [17], and analytic hierarchy process [18]. Also, quantitative methods have been proposed using heuristic methods [19], simulation techniques [20], and analytical methods [2].

Seiti et al. [21] used a multi-criteria decision-making (MCDM) method based on fuzzy probability and D number to evaluate the breakdown risk of a production line. The MCDM provides a risk rank of a machine breakdown from high to low, which is useful for decision making. However, the model highly relies on expert knowledge, and the result of the prediction is subjective to the risk grade given by the expert. 

Bayesian network (BN) is an effective algorithm to handle complex systems like production lines, and the possibility of applying BN in production line lifetime prediction is investigated by Wang et al. [22]. A simulation model is also an option for lifetime prediction, and a study showed that the stochastic simulation model achieved high accuracy in predicting the lifetime of a rectifier system [11]. The drawback of the simulation model is that it requires expert knowledge to build. 

Among approaches used for PdM, machine learning-based ones are considered to be the most suitable approaches because they can handle high-dimensional problems that consist of hundreds or thousands of variables such as voltages, flows, and currents [23]. There exist two main categories of machine learning-based techniques for PdM. The first one is supervised approaches where the failure information exists in the dataset. The second one is unsupervised approaches, where there is only process information, and no failure-related information exists [23].

Machine learning methods have been increasingly applied in different areas to perform various tasks. Fault diagnosis is the most common application area of machine learning, which determines whether to send equipment to fix (or to be replaced). This kind of task mainly uses binary classification or multi-category classification algorithms to predict failures or malfunctions. Luo and Wang [24] applied random forest to identify the malfunction of robot arms by learning patterns from the torque sensors. However, there is a lack of models to predict the remaining lifetime of a machine because there are not enough indicators to measure the health status of a machine [25]. Also, the health status is largely affected by the operating environment, and some failure is caused by accident rather than deterioration.

As a result of the wide adoption of machine learning techniques in many application fields, recently, researchers focused on the use of machine learning techniques for predicting the machine lifetime. RUL is mostly used as a risk indicator for preventive maintenance service. It indicates how long a machine can operate as usual before the breakdown. RUL modeling requires a run-to-failure dataset from the operation of machines, which is difficult to acquire.

A set of turbo engine run-to-failure datasets is provided by NASA [26], and the data is used in many research papers to predict the RUL. Ramasso and Saxena [27] published a survey on prognostic methods used for the NASA turbo engine datasets and divided the prognostic approaches into three categories. The first category is the use of functional mappings between the set of inputs and RUL. For the first category, they reported that the dominant underlying machine learning algorithm is artificial neural networks (ANNs). The second category of techniques is the functional mapping between the health index and RUL. The third category is similarity-based matching techniques. Benchmarking of prognostic methods has been conducted on the NASA turbo engine dataset, and it was shown that most of the studies use a health index to map between input features and the RUL [27].

Research on RUL prediction can be divided into the following categories [28]: knowledge-based models (KBM), physical models (PM), data-driven models (DDM), and deep learning (DL). In knowledge-based models, experts define the rule sets and evaluate the condition of the equipment based on previous failures and sometimes they result in contradictions [29]. Physical models model the complete equipment, however, it is expensive and not always achievable. Early data-driven models used statistical and stochastic approaches such as Markov models, proportional hazard modelling, and Bayesian techniques with Kalman filters [30]. There is an assumption of stochastic models. Identical components are considered to be statistically identical and random variables are independent. However, this assumption is not true for some datasets that include random starting conditions. In general, statistical approaches form a hypothesis before building the model and work on several assumptions. Each statistical approach comes with a different assumption. However, in machine learning, algorithms are run on data directly. Also, machine learning-based approaches often provide better performance than statistical approaches in terms of accuracy though more computational power is required in recent years. To avoid the limitations of statistical and stochastic models and achieve higher performance, we aimed to focus on machine learning approaches instead of developing models using statistical methods.

Ahmadzadeh and Lundberg [31] published a review article in 2013 and categorized the RUL prediction studies into the following four categories:

- Physics-based: Physical model, cumulative damage, hazard rate, proportional hazard rate, nonlinear dynamics
- Experimental-based
- Data-driven: Neural network (NN), support vector machine, Bayesian network, hidden (Markov, semi-Markov)
- Hybrid: Statistical model, Fourier transform with NN, statistical model with NN, fuzzy logic with NN, wavelet transform analysis with a statistical model, dynamic wavelet with NN.

They also provided the advantages and disadvantages of these approaches. Physics-based approaches are computationally expensive, too stochastic to model the fault, and need the evaluation of assumptions. Experimental approaches are costly and required to verify the theoretical models. Data-driven approaches use nonlinear relationships and perform pattern recognition. Hybrid methods combine two methodologies such as statistical methods and neural networks. They reported that the best model is selected based on the availability of the data [31].

Okoh et al. [32] classified studies into the following RUL prediction methodology types in 2014: Model-based, analytical-based, knowledge-based, and hybrid. Also, they presented the following prediction technique types: Statistics, experience, computational intelligence (CI), physics-of-failure, and fusion. Artificial neural networks are represented under the CI category. They also presented in a table that while statistics-based approaches are unable to process large datasets, CI techniques are able to handle large datasets.

Hu et al. [33] categorized RUL prediction techniques into the following three categories: Physics-based approaches, data-driven techniques (probabilistic, artificial intelligence methods, and stochastic), and hybrid approaches that combine the physics-based and data-driven techniques. They proposed the state space model (SSM) for RUL prediction.

Si et al. [34] reviewed statistical data-driven approaches and categorized the condition monitoring (CM) data into direct CM data and indirect CM data. Under the direct CM data, the following approaches were presented: Regression-based, Wiener process, gamma process, and Markovian-based. Under the indirect CM data, the following techniques were provided: Stochastic filtering-based, covariate-based hazard, and hidden Markov model & hidden semi-Markov model-based. They discussed several challenges related to the RUL prediction. For example, they stated that a new RUL prediction model is required in the case of very limited observed failure data or no observed failure data because statistical models cannot be used in these two cases.

Djeziri et al. [35] categorized the RUL estimation approaches into the following categories:

- Expert model-based: Expert models, fuzzy logic
- Data-driven approaches
  ○ Trend modeling methods: Machine learning, statistical models, stochastic models, deterministic models, probabilistic models
  ○ Machine Learning
- Model-based approaches: Specific degradation models

As we see in these six review articles [28,31,32,33,34,35], different researchers categorized RUL prediction studies into different categories, however, main techniques, namely machine learning, statistical models, physics-based techniques, hybrid approaches, and knowledge/expert-based approaches often appear in these taxonomies.

Recently, deep learning-based RUL prediction models have been proposed. Li et al. [36] developed a multi-scale deep convolutional neural network (MS-DCNN) and used the min-max normalization with the MS-DCNN algorithm for RUL prediction. They compared the performance of their model with other state-of-the-art models and showed that the new model provides promising results on the NASA C-MAPSS dataset. 

Hou et al. [37] developed a deep supervised learning approach using similarity to improve the prediction performance. Since the health indicator (HI) construction techniques depend on manual labeling or expert opinion, Hou et al. [37] also developed an unsupervised learning approach-based on restricted Boltzmann machine (RBM) to construct the HI. They showed that their performance provides superior performance compared to the other traditional approaches. Cheng et al. [38] proposed a transferable convolutional neural network (TCNN) to learn domain invariant features for bearing RUL prediction. They showed that their model avoids the influence of kernel selection and present a better performance for RUL prediction. Wang et al. [39] proposed a recurrent convolutional neural network (RCNN) for RUL prediction and demonstrated its effectiveness based on two case studies. They showed that the proposed model can predict the RUL prediction of rolling element bearings and milling cutters effectively. Their model provides a probabilistic result for RUL prediction and simplifies decision making. Chen et al. [40] developed a recurrent neural network (RNN) model using an encoder-decoder structure with an attention mechanism for RUL prediction of rolling bearings. They showed that their model can work with little prior knowledge and provides better performance than the other models. Wu et al. [41] proposed a deep long short-term memory (DLSTM) network that uses the grid search strategy for RUL prediction. They demonstrated that this model provides satisfactory performance for the RUL prediction of turbofan engines. Li et al. [42] applied the generative adversarial network (GAN) algorithm to compute the distribution of the healthy state data and proposed a health indicator. Promising results were achieved on two rotating machinery datasets. Su et al. [43] integrated the variational autoencoder (VAE) algorithm with a time-window-based sequence neural network (twSNN) for RUL prediction and demonstrated the effectiveness of their model on a dataset of aircraft turbine engines.

While deep learning-based models can provide better performance for RUL prediction, there are several limitations of using this type of algorithms. For instance, they need a lot of data, hyperparameter tuning is required, and there is a high computational cost. To avoid these limitations, in this study we aimed to develop a novel machine learning model that is still accurate but does not consist of these limitations. 

In our study, MLP that is a special ANN topology, along with other machine learning methods (i.e., grid search parameter optimization, normalization, and feature selection), are combined with the interpolation technique, and a novel machine learning-based RUL prediction model is built. The effectiveness of this new model is investigated on NASA turbo engine datasets.

## 3. Data Analysis

There are four turbofan datasets running on different conditions, as shown in Table 1. For instance, the dataset FD001 has 100 turbo engine units running under one condition with only a high-pressure cylinder (HPC) fault. In the training dataset, the turbo running from a certain point to failure while in the testing dataset, the records stop at a middle point. The task is to predict the RUL of the turbofan in the testing dataset. In other words, an algorithm needs to predict when the turbo will break, and the required maintenance is needed. In Table 2, the data structure of the training dataset is presented, and in Table 3, the data structure of the RUL dataset is shown. Settings data and sensory data are all anonymous data.

**Figure 1:**
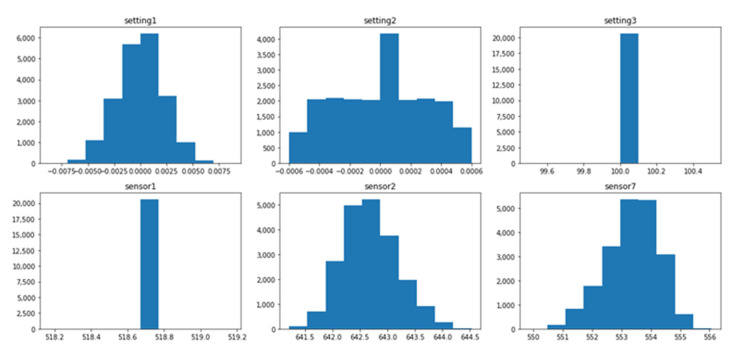
FD001_Train—distribution of selected variables.

**Figure 2:**
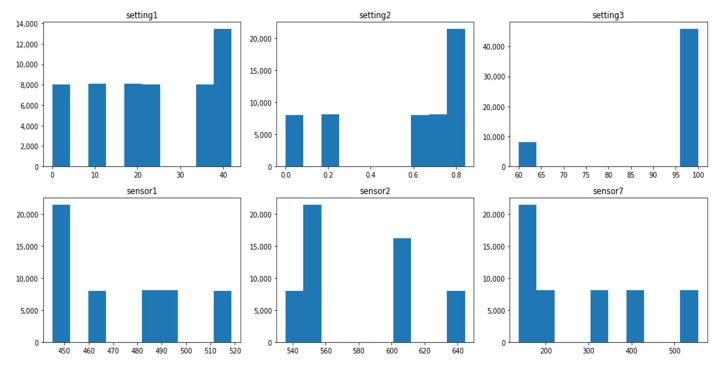
FD002_Train—distribution of selected variables.

**Figure 3:**
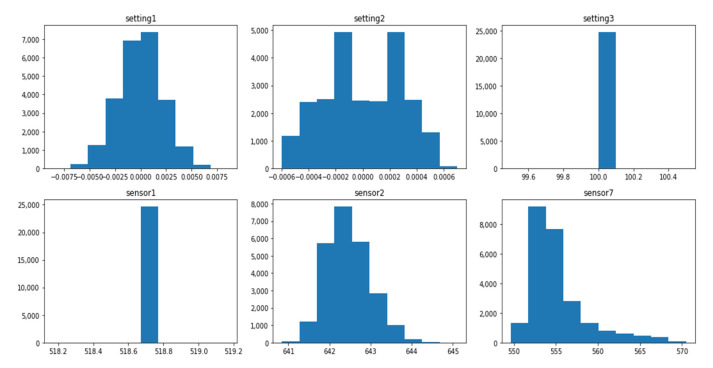
FD003_Train—distribution of selected variables.

**Figure 4:**
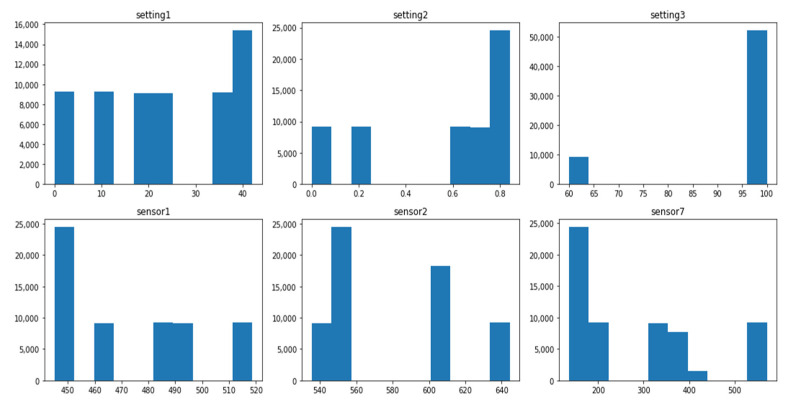
FD004_Train—distribution of selected variables.

The data distribution of each dataset is different, and this difference is associated with several operating conditions and fault modes, as shown in Table 1. The setting variables and sensor variables can be constant, discrete, and continuous. The same variable can have different data distribution forms in different datasets. For instance, the variable setting1 is a continuous variable with normal distribution in the dataset FD001_train, as shown in Figure 1, while it is a discrete variable distributed on six values in the dataset FD002_train, as shown in Figure 2. Similarly, the variable sensor1 changes from a constant value in the FD003_train, as shown in Figure 3 to a discrete distribution in the dataset FD004_train, as shown in Figure 4. In general, features of dataset FD001 and FD003 are mostly continuously distributed while the features of FD002 and FD004 are discretely distributed.

The value range of each feature varies significantly within the same dataset. In the dataset FD001_train, setting1 ranges from −0.087 to 0.087, while sensor7 ranges from 549 to 556, as shown in Figure 1. Additionally, the range of the same variable changes dramatically in different datasets. The range of setting 1 in FDD02 & FD004_train is (0–40), as shown in Figure 2 and Figure 4 while the range in FD001 and FD003 is (−0.087–0.087) as shown in Figure 1 and Figure 3. This can be explained by the fact that FD001 has only one operation condition, while FD002 has six different conditions. 

**Figure 5:**
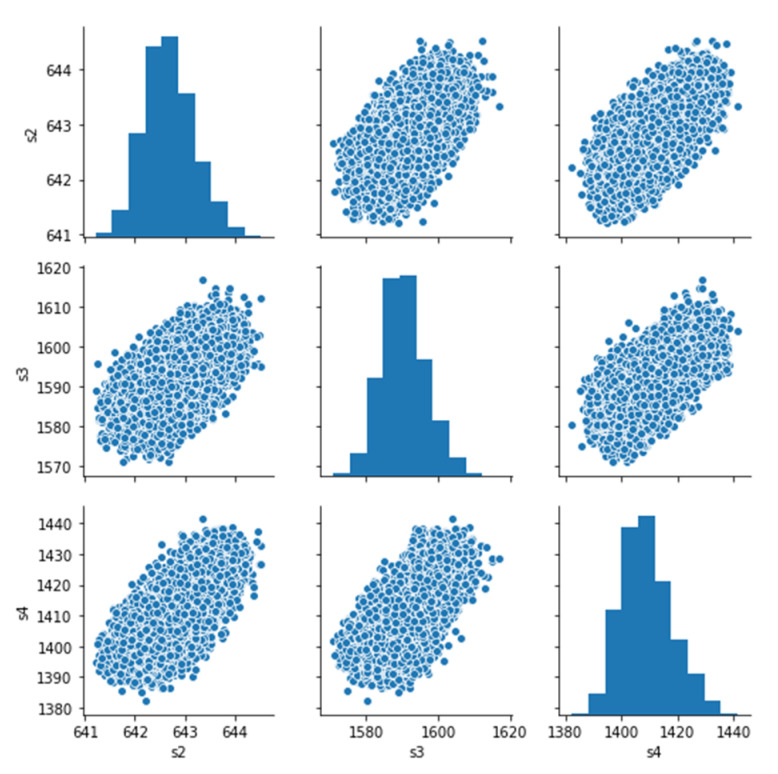
Correlation plot of variables (sensor2, sensor3, and sensor4) of FD001.

**Figure 6:**
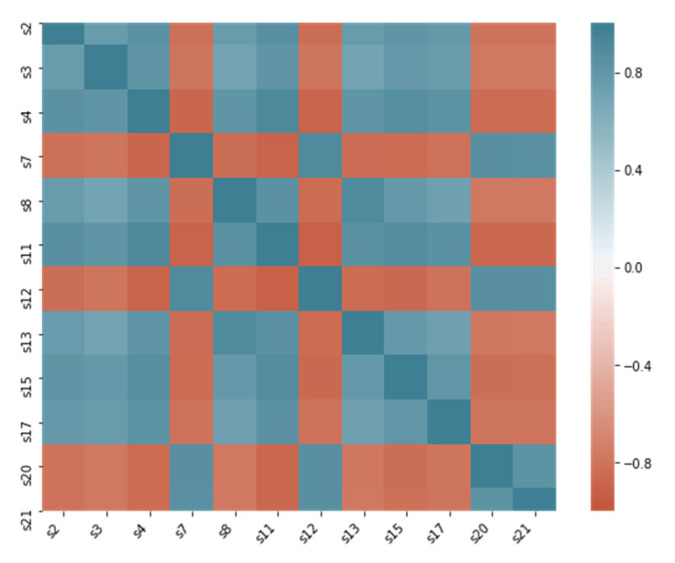
Correlation heatmap of most correlated variable pairs.

According to our observations, the setting variables’ distributions reflect the operation condition of each dataset. Datasets FD001 and FD003 operate under the same condition and have similar setting variable value distribution. FD002 and FD004 operate under six different conditions, and their variable value distributions are similar. The correlations between features are also strong. Figure 5 presents the correlation plot of variables (sensor2, sensor3, and sensor4) of the FD001dataset., Figure 6 shows that some sensors are highly correlated.

## 4. Methodology

**Figure 7:**
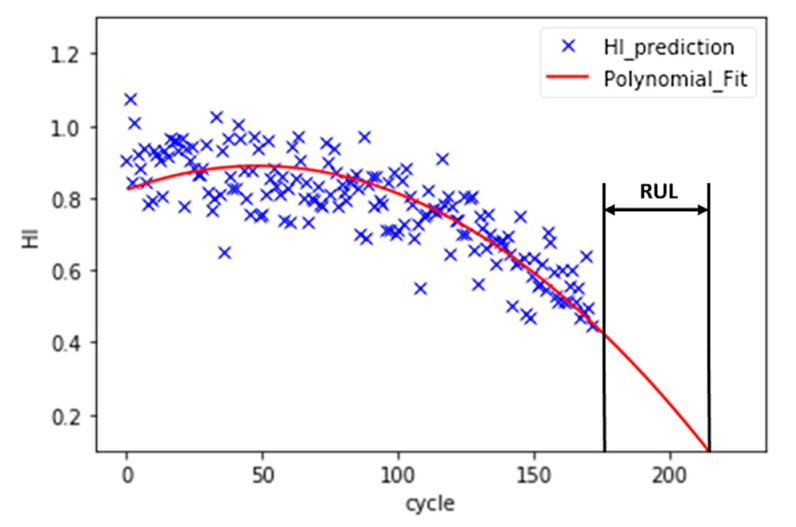
The HI prediction and RUL estimation for FD001_test turbo engine unit 84.

Instead of direct measurements of RUL, usually, indirect measures are adopted. For this reason, the concept of the health index (HI) is often used to estimate the RUL [44]. Instead of directly predicting the RUL, a machine learning model is trained to predict the HI of a turbo engine in each cycle. Since the RUL is not provided in training datasets, the use of supervised learning approaches to predict the RUL label is not possible. Then, a polynomial function is fitted to HIs, and the interception between the polynomial and cycle axis is the failure point. In Figure 7, this approach and the calculation of RUL is represented.

In the training datasets, each turbo machine runs from good health conditions to failure one. Thus, this research assumes the HI of initial cycles is maximum and the HI of last cycles is minimum. Therefore, we can assume that N initial cycles that have HI = 1, and N last cycles that have HI = 0. Then, the rest of the data points label can be estimated by interpolation. After the interpolation, all points are labeled and supervised learning can be applied.

**Figure 8:**
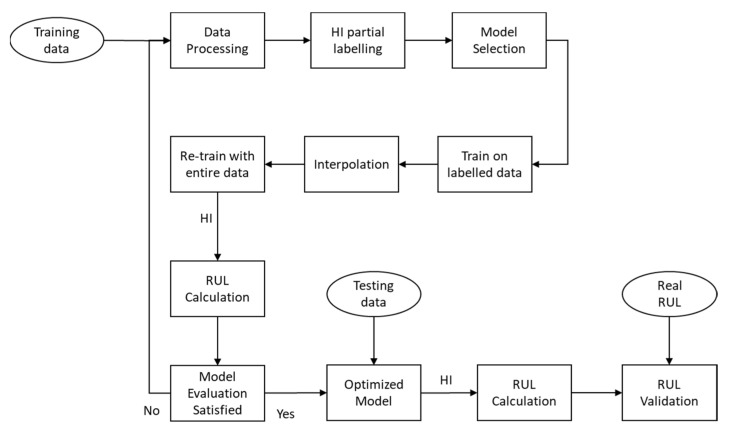
Flowchart of our RUL prediction approach.

Figure 8 represents the flowchart of our interpolation and machine learning-based prediction model. First, a model is trained with partially labeled data. Then, the trained model is used to interpolate the rest of the unlabeled training points with HIs. After the interpolation, the entire HI labeled dataset is used as a feedback mechanism for the model to re-train the model. 

### 4.1. Data Pre-Processing

Some features are constant in the dataset, and thus, their variance is zero. All zero variance variables are removed before the training stage because they do not contain useful information for machine learning. Since the value range is substantially different in different variables, it can be difficult to find the optimal point for the cost function. It also tends to take a long time to reach the optimum, which uses extra computational power. Therefore, the training and testing datasets need to be normalized. There are two widely used methods for normalization, which are Z-scores (Equation (1)) and min-max-scale (Equation (2)). Both methods are applied, and the one with the best evaluation result is selected.

(1) $$x^{\prime }=\;\frac{x-\mathrm{mean}\left(x\right)}{\mathrm{std}\left(x\right)}$$

(2) $$x^{\prime }=\;\frac{x-x_{\min}}{x_{\max}-x_{\min}}$$

The correlation heatmap shown in Figure 6 indicates that half of the features in the dataset are highly correlated to each other. For avoiding the negative effect of the covariance, principle components analysis (PCA) is applied for features in the dataset. The number of PCA components equals the number of features to catch all the variance in the original data.

### 4.2. Model Selection

Two different approaches have been applied during the learning process. The first model learns from the part of the labeled dataset and conducts the interpolation for the rest of the data points. The second model learns from the entire dataset, and it uses the final model to predict the HI. For the first model, several algorithms were applied, and the linear regression (LR) achieved the best interpolation results. The results of other algorithms do not show a regular degradation trend, and as such, it is difficult to fit with a polynomial curve. Therefore, a linear regression model is selected to be the first model. 

According to our literature search in electronic databases, multi-layer perceptron neural network (MLP), random forest (RF) [45], and support vector regression (SVR) algorithms are the three most used algorithms for the PdM category. Therefore, MLP, RF, SVR, and LR are applied as the second model to perform the re-training process of the entire dataset.

The grid search cross-validation method is applied to find the best hyperparameters of RF and SVR. The best hyperparameters have the lowest HI MSE. For the RF, parameters are selected as follows: estimator = 100 and depth of tree = 6. For the SVR, the radial basis function kernel is chosen, and gamma is assigned to 0.1. For the MLP model, units and cycles are excluded from the inputs, and Table 4 shows the parameters used in this study.

### 4.3. Interpolation and Model Training

**Figure 9:**
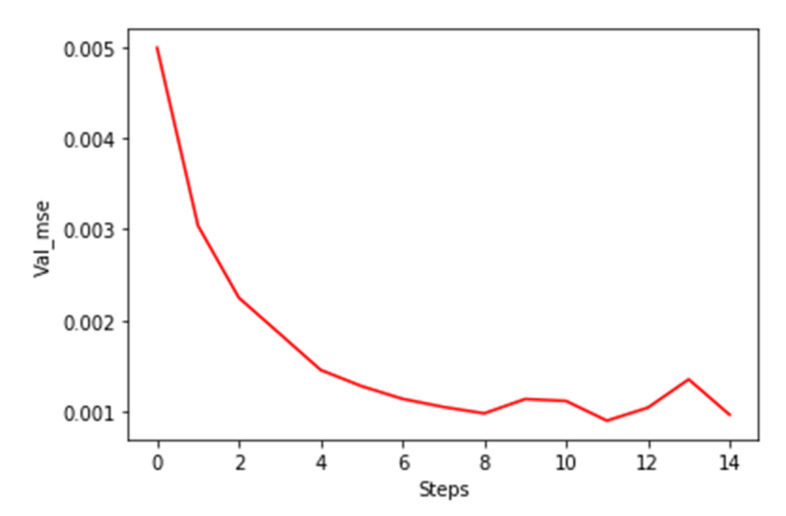
Early stop at Val_mse = 0.001 by monitoring validation MSE.

There are three stages in the interpolation and re-train process, which are partial dataset training, interpolation, and full dataset training, as shown in Figure 8. After the labeling process, a part of the dataset is labeled with the HI index, which can be used for supervised learning. The trained model then predicts the rest of the unlabeled data points so that the whole dataset is labeled with HI. Last, the model is re-trained with the entire labeled dataset to improve the mean squared error (MSE). 5-fold cross-validation is used to prevent overfitting in both the first and second stages. The training process stops earlier if the validation MSE stops decreasing in the next five steps, as shown in Figure 9.

**Figure 10:**
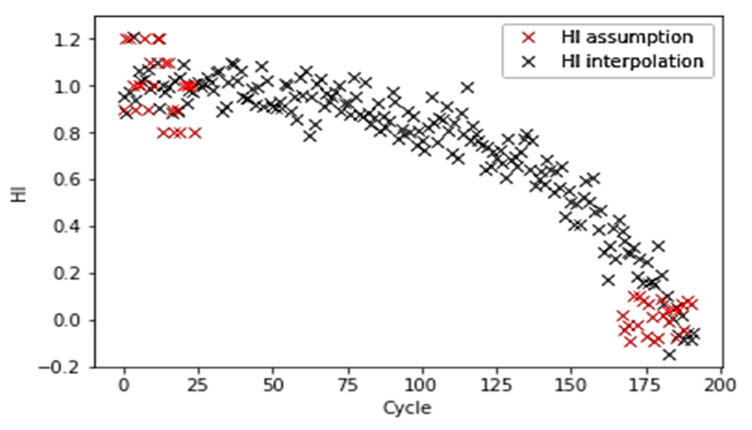
Linear interpolation by partial marked HI points.

**Figure 11:**
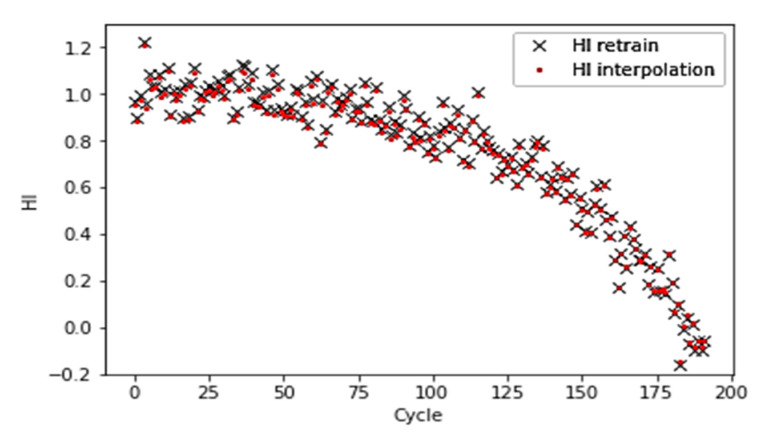
Retraining of the interpolated HI with a selected model (correlation coefficient r = 0.999).

Figure 10. demonstrates the interpolation process of HI with partially labeled data points. Figure 11 shows that a selected model learns from the entire dataset after the interpolation and predicts a similar HI pattern for each turbo unit.

### 4.4. Evaluation

Because the purpose of the machine learning model is to predict the RUL instead of the HI, the MSE of the training cannot be used to evaluate the performance of the model. A model may have very low training MSE, but it may have a high deviation in the RUL prediction. In other words, the tuning of hyperparameters, such as the size of N points, the number of PCA components, and model parameters, cannot rely on the training MSE. According to Figure 7, the estimation of RUL is based on a polynomial curve fit. Therefore, a second-order polynomial (Equation (3)) is fitted to the HI. The coefficient must be a negative number to ensure that the curve is decreasing.

(3) $$y={\mathrm{ax}}^{2}+\mathrm{bx}+c$$

(4) $${\mathrm{MSE}}_{\mathrm{RUL}}=\frac{1}{n}\overset{n}{\underset{i=1}{\sum }}\left(T_{r}-T_{p}\right)$$

(5) $${\mathrm{MSE}}_{{\mathrm{RUL}}_{\mathrm{Val}}}=\frac{1}{n}\overset{n}{\underset{i=1}{\sum }}\left({\mathrm{RUL}}_{r}-{\mathrm{RUL}}_{p}\right)$$

**Figure 12:**
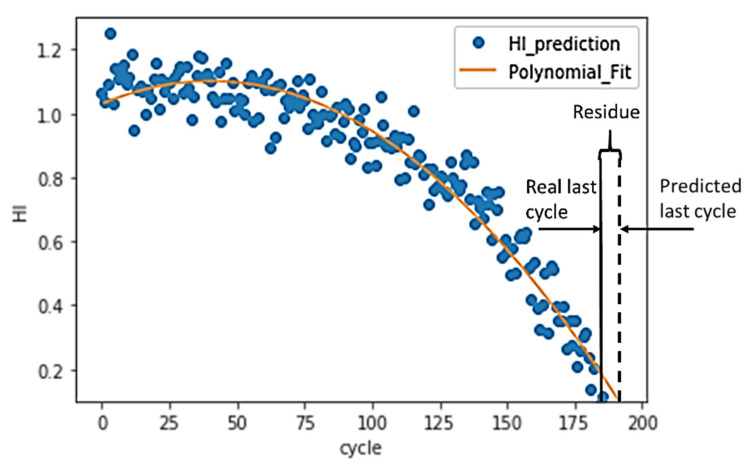
Accuracy of RUL prediction.

In the training dataset, the turbo engines run from healthy conditions to failure one. Therefore, the RUL at the last cycle of the training data point should be zero. The residue between the real last cycle and the predicted last cycle can be calculated, as shown in Figure 12. The RUL MSE can then be calculated based on Equation (4). The n is equal to the number of turbo units in the dataset. T_r_ and T_p_ stand for the real last cycle and the predicted last cycle, respectively. 

The model is optimized against the MSE_RUL_ by tuning hyperparameters. The model setting with the lowest MSE_RUL_ is used for validation. After training, the model needs to be validated with the testing dataset. The testing data is processed with the steps, as in the case of training data. The testing data should have the same variables as the training data, and it is normalized with the training data mean and variance if Z-scores are performed. The testing data is transferred to PCA components with the training data eigenvector matrix.

Then, the processed testing data is fed to the model to predict HI for each cycle. A second-order polynomial is fitted to the HIs with the minimum MSE. The interception between the curve and the cycle axis is the prediction of the end cycle. RUL can be calculated to subtract the last cycle of the test data from the end cycle. The MSE_RUL_Val_ can be calculated based on Equation (5). The n equals the number of turbo units in the dataset. RULr and RULp stand for the real RUL of the test data and the predicted RUL, respectively.

## 5. Experimental Results

**Figure 13:**
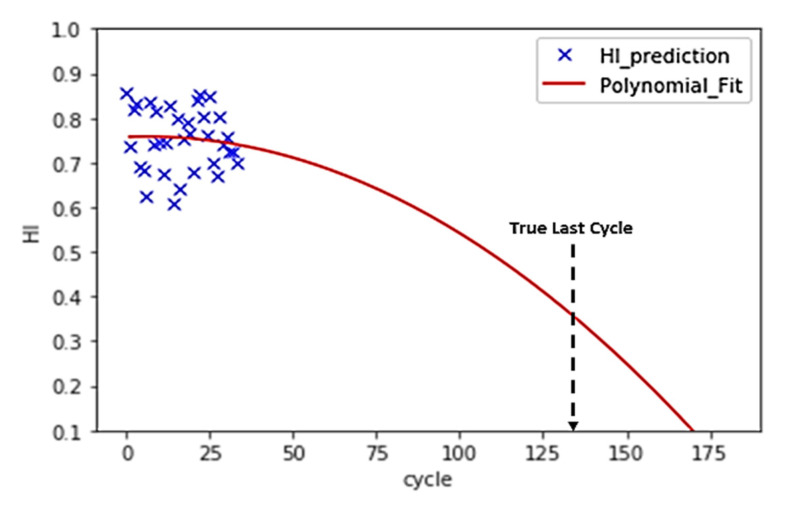
FD001_Test turbo unit 85 (34 cycles) test data validation.

**Figure 14:**
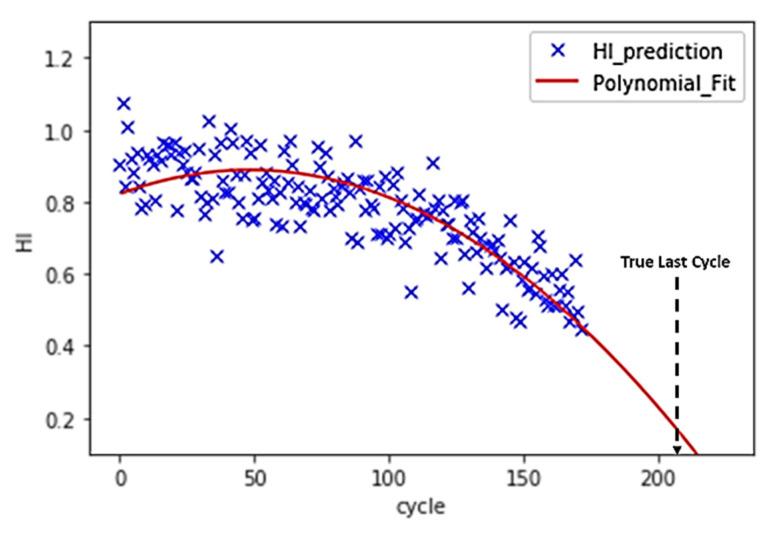
FD001_Test turbo unit 84 (172 cycles) test data validation.

**Figure 15:**
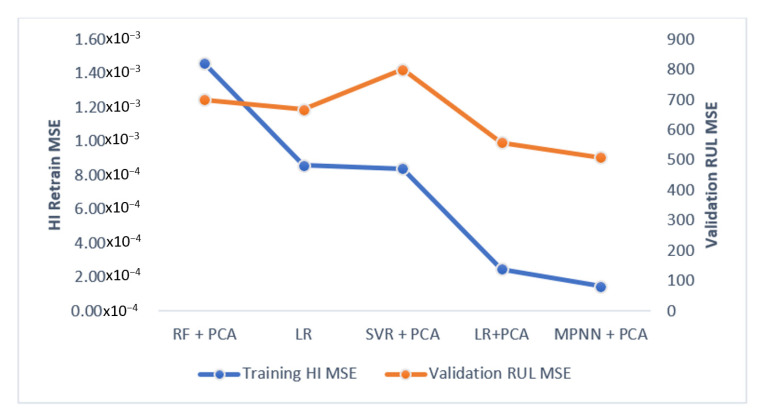
Result of FD001- the correlation between the HI MSE and the RUL validation MSE.

A dataset with multiple fault modes and multiple operation conditions achieved higher HI training MSE than the dataset with single fault mode and single operating condition. The re-training of the interpolated data improves the HI MSE in all algorithms, as shown in Table 5. Similar to the HI MSE, dataset FD004, which has the most complex data composition, has the highest RUL training MSE, whereas FD001 has the lowest RUL training MSE for all algorithms. Validation RUL MSE has the same pattern as the training RUL MSE, where the MSE increases as the data becomes more complex. Validation MSE of units with a cycle greater than 100 tends to have a lower value compared with the validation MSE of the unit with all cycles. Figure 13 and Figure 14 show a comparison between short cycle prediction and long cycle prediction. The unit 84 prediction accuracy outperforms the unit 85 prediction because more information is provided in unit 84. In Figure 15, the correlation between the HI MSE and the RUL validation MSE is presented.

Table 5A,B show that LR with PCA has a lower HI training MSE and RUL validation MSE compared to the LR without PCA. Particularly, the MSE drop is significant for the FD001 and FD002, which have simpler data composition. The MSE does not change obviously for the datasets FD003 and FD004, which are multi-fault modes and multi-operation conditions.

By comparing these results, we have noticed that the MLP algorithm provides the lowest RUL validation MSE. Also, MLP has lower HI MSE than the other algorithms. It has a notably better result in the prediction of FD001, which has a single fault mode and a single condition.

## 6. Discussion

### 6.1. Main Discussion

By assuming initial points with HI = 1 and endpoints with HI = 0, a linear interpolation of the rest of the points HI assigns labels to all samples. Then, the use of supervised learning approaches becomes possible, and the machine learning model can learn from the whole dataset. The interpolation process, as demonstrated in Figure 12, shows a clear trend line of the turbo deterioration. The matched trend line predicts the RUL accurately with a small training RUL MSE, as shown in Table 5. The positive correlation among the training HI MSE, training RUL MSE, and the validation RUL MSE prove that the interpolation and training process is valid and effective. 

The results show that PCA is an effective data pre-processing algorithm to improve prediction accuracy. First, PCA can reduce the adverse impact of noise in the dataset. Second, the dataset is highly correlated, and correlated feature pairs can lead to lower prediction accuracy. PCA can avoid the effect of covariance and correlation by transferring data into a new space where all components are orthogonal to each other. The data is transferred to an equal number of PCA components to catch all original variances, and all PCA components are independent of each other. 

The result shows that the MLP-based prediction model provides the best performance in predicting the RUL. It has a significantly better performance compared to the RF and SVR. However, the validation RUL MSE of MLP does not significantly outperform the LR. A small dataset might compromise the performance of MLP. The neural network-based model has higher performance on a large training dataset, whereas the training dataset size in this case study is relatively limited. In the scenario of the production lines, MLP may have higher performance as more data is available, and the task is more complex. The accuracy of the prediction is largely affected by the dataset type. In all predictions, the performance on FD001 outperforms the performance on FD002. FD001 has only data from the sea level operational condition, while the data of FD002 is from six different operational conditions. However, the FD002 data size is only 150% greater than the size of FD001. Consequently, there are fewer training points for each condition in FD002 than the FD001. Hence, FD001 has a smaller HI MSE and RUL MSE than that of the FD002. According to the result shown in Table 5, datasets FD001 and FD002 have a better overall validation RUL prediction accuracy compared to the datasets FD003 and FD004 for all models. The low performance on FD003 and FD004 might be largely related to the mix of fault modes in these two datasets. In the data analysis, there are two fault modes, namely HPC fault, and Fan fault. The HPC fault may be independent of the turbofan fault, and whichever part fail may lead to the turbo failure. Therefore, the ideal way of RUL prediction is to predict each fail mode separately and see which part fails first. Two independent health indexes should be used for each working mode. However, the training datasets only have single fault mode training data for HPC, and there is no separate fault mode training data for the fan fault. 

The validation RUL MSE for cycle >100 has a significantly lower value than that of the full-cycle units, which contain units with cycle <100 because fewer cycles unit provides less information to the model to judge its HI development trend. The HI degradation is insignificant at the early stage, and the degradation accelerates as the cycle grows larger. Hence, units with more cycles tend to have more obvious degradation patterns for the polynomial to fit and to predict the RUL. For instance, the turbo unit 85, as shown in Figure 13 of FD001_Test with 34 cycles, has a much higher RUL prediction error than that of unit 84, as shown in Figure 14 in the same dataset. 

**Figure 16:**
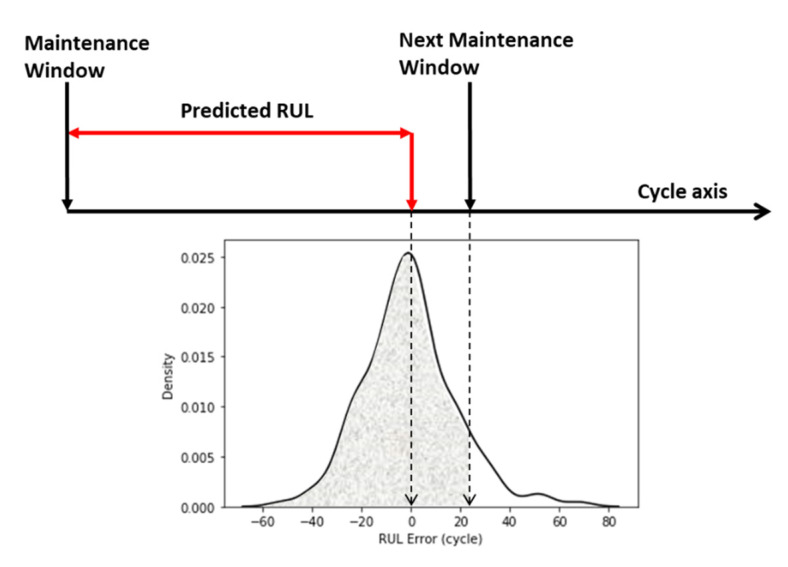
The RUL error distribution and confidence interval for maintenance.

In the scenario of production line risk management, the machine learning-based RUL prediction can help managers to evaluate the possibility of a machine failure before a maintenance window. In large scale manufacturing, the maintenance time is fixed, and multiple machines are maintained in one maintenance window. It is not possible and economical to maintain all machines in one window. Thus, the manager needs to decide on which machines to conduct the maintenance in the scheduled maintenance window. The machine learning-based RUL model can generate a density chart of RUL prediction error. To this end, the manager can estimate the possibility of a machine to fail before the next maintenance window, as shown in Figure 16. The management team can decide whether to add a machine to the current maintenance list or leave it to the next maintenance window.

Although several deep learning-based models have been developed for RUL prediction recently, they have some limitations such as requiring a lot of data for training, the difficulty of hyperparameter tuning, and high computation cost. Since our aim was to build a model that can work even with limited data, we did not focus on deep learning algorithms in this research. However, as part of new research, we can compare the performance of our model with the recently developed deep learning-based RUL prediction models. Since our focus was not the use of deep learning, we did not compare our results with models that utilize deep learning.

### 6.2. Threats to Validity

This study aims to develop an effective way of predictive maintenance tasks by using machine learning algorithms. A limited number of machine learning algorithms, pre-processing methods, and parameter optimization techniques are evaluated. The performance of this machine learning-based prediction model can be further improved by other advanced machine learning techniques. For instance, some researchers apply machine learning directly to predict the RUL instead of mapping to a HI. Furthermore, feature information, such as the name and properties, are not fully explained in the public datasets. Thus, all features are treated equivalently in our study, whereas in the real situation, experts can filter out some irrelevant features based on the available information of features. This extra time can save a lot of work for data processing, and improve the prediction accuracy.

The machine learning approaches adopted in this study work better with a single fault working mode dataset. The performance of the multi-fault modes dataset is not accurate enough for real practice. As such, the machine learning-based model proposed in this paper requires the single fault mode training data to perform effectively. Also, the data size of this study is limited, which restricts the applicability of the machine learning model in a broader context. In the real production line scenario, the data size is larger because a large amount of data is generated continuously, and the single fault mode data is difficult to achieve. Generally, data generated from the production line contains malfunction signals from different components. Extra data analysis may need to be taken to extract data for a single fault mode, which is expensive and time-consuming. More research is needed to be conducted to improve the performance of the machine learning-based model with multi-fault modes of training data.

## 7. Conclusions

In this article, we have provided a machine learning-based predictive maintenance approach for production lines. We have applied the production line case study for turbo engines. This study on the turbo engine RUL prediction demonstrates the possibility of using interpolation and machine learning algorithms to predict the RUL of production lines. The interpolation method can effectively map the relationship between features and the RUL, and the MLP-based prediction model provides the best performance in predicting RUL from the interpolated HI. The proposed model applies normalization and feature selection techniques (i.e., principle component analysis) during the pre-processing stage, utilizes from interpolation, uses grid search for parameter optimization, and is built with multilayer perceptron neural network (MLP) machine learning algorithm. Our novel model has been implemented and evaluated to predict the remaining useful life (RUL) of engines on NASA turbo engine datasets. The result of the prediction provides useful guidance to the management to conduct proactive maintenance before production line failure. Experimental results demonstrate that the performance of our proposed model is remarkable in predicting the RUL of turbo engines, and predictive maintenance is beneficial.

The performance of machine learning for RUL prediction is primarily affected by the data property, including size, dimension, noise level, fault modes, and environmental variation. Training data with a single fault mode and single operation environment can improve the RUL prediction significantly; however, acquiring single environment data is difficult in the real production environment.

## Figures and Tables

**Table 1:** Datasets.

Training Dataset		Testing Dataset				# of Conditions Engine	Fault Mode
Dataset	Dimension	Dataset	Dimension	Dataset	Dimension		
FD001_train	20,631 × 26	FD001_test	13,096 × 26	RUL1	100 × 2	1	HPC Degradation
FD002_train	53,759 × 26	FD002_test	33,991 × 26	RUL2	259 × 2	6	HPC Degradation
FD003_train	24,720 × 26	FD003_test	16,596 × 26	RUL3	100 × 2	1	HPC & Fan Degradation
FD004_train	61,249 × 26	FD004_test	41,214 × 26	RUL4	248 × 2	6	HPC & Fan Degradation

**Table 2:** The data structures of the training and testing datasets.

Unit	Cycle	Setting1	Setting2	Setting3	Sensor1	……	Sensor21
Int	Int	Float	Float	Float	Float	Float	Float

**Table 3:** The data structure of the RUL dataset.

Unit	RUL
Int	Int

**Table 4:** MLP model setup.

	Connection	Number of Units	Input Dimension	Activation Fun
**Input Layer**	Dense	24	24	Tanh
**Hidden Layer-1**	Dense	20	-	Tanh
**Hidden Layer-2**	Dense	5	-	Tanh
**Output Layer**	Dense	1	-	Linear
	**Loss function**	**Optimizer**	**Learning Rate**	**Belta_1**
**Compiling**	MSE	Adam	3 × 10^−5^	0.9

**Table 5:** (**A**) Linear Regression, (**B**) Linear Regression + PCA, (**C**) MLP + PCA, (**D**) RF + PCA, (**E**) SVR + PCA; HI Training MSE stands for the MSE of the partial data training; HI Retrain MSE stands for the MSE of the retrain on the whole dataset; Training RUL MSE represents the evaluation of RUL with the training data; Validation RUL MSE represents the validation result of RUL estimation of the test data; Validation RUL MSE represents the validation of RUL in test data with cycle > 100.

Dataset	HI Training MSE	HI Retrain MSE	Training RUL MSE	Validation RUL_MSE	Validation RUL_MSE (cycle > 100)
FD001	3.18 × 10^−3^	8.60 × 10^−4^	20	668	499
FD002	3.87 × 10^−2^	2.90 × 10^−3^	26	1031	390
FD003	3.57 × 10^−2^	7.66 × 10^−4^	32	1332	1162
FD004	5.88 × 10^−2^	2.20 × 10^−3^	149	2181	1108
(**A**)					
FD001	3.71 × 10^−2^	2.48 × 10^−4^	21	558	468
FD002	3.61 × 10^−2^	4.41 × 10^−4^	36	748	358
FD003	3.34 × 10^−2^	2.31 × 10^−4^	35	1387	1186
FD004	4.07 × 10^−2^	5.38 × 10^−4^	94	1904	1094
(**B**)					
FD001	3.65 × 10^−2^	1.47 × 10^−4^	55	509	504
FD002	3.62 × 10^−2^	1.92 × 10^−4^	43	746	364
FD003	3.36 × 10^−2^	9.69 × 10^−5^	21	1259	1100
FD004	4.25 × 10^−2^	8.56 × 10^−5^	94	1427	1031
(**C**)					
FD001	3.69 × 10^−2^	1.46 × 10^−3^	18	701	511
FD002	3.60 × 10^−2^	4.40 × 10^−3^	21	857	436
FD003	3.37 × 10^−2^	3.56 × 10^−3^	136	1895	1411
FD004	4.09 × 10^−2^	1.05 × 10^−2^	316	1994	1613
(**D**)					
FD001	3.70 × 10^−2^	8.40 × 10^−4^	71	800	568
FD002	3.62 × 10^−2^	6.10 × 10^−3^	23	776	382
FD003	3.36 × 10^−2^	1.55 × 10^−3^	85	1089	947
FD004	4.29 × 10^−2^	1.01 × 10^−3^	162	1575	1199
(**E**)					

## Data Availability

Not applicable.
